# The efficacy of stereotactic body radiation therapy on huge hepatocellular carcinoma unsuitable for other local modalities

**DOI:** 10.1186/1748-717X-9-120

**Published:** 2014-05-28

**Authors:** Jenny Y Que, Li-Ching Lin, Kuei-Li Lin, Chia-Hui Lin, Yu-Wei Lin, Ching-Chieh Yang

**Affiliations:** 1 Department of Radiation Oncology, Chi Mei Medical Center, No.901, Zhonghua Road, Yongkang district, Tainan, Taiwan

**Keywords:** Huge, Hepatocellular carcinoma, Stereotactic body radiation therapy

## Abstract

**Background and aim:**

To evaluate the safety and efficacy of Cyberknife stereotactic body radiation therapy (SBRT) and its effect on survival in patients with unresectable huge hepatocellular carcinoma (HCC) unsuitable of other standard treatment option.

**Methods:**

Between 2009 and 2011, 22 patients with unresectable huge HCC (≧10 cm) were treated with SBRT. dose ranged from 26 Gy to 40 Gy in five fractions. Overall survival (OS) and disease-progression free survival (DPFS) were determined by Kaplan-Meier analysis. Tumor response and toxicities were also assessed.

**Results:**

After a median follow-up of 11.5 month (range 2–46 months). The objective response rate was achieved in 86.3% (complete response (CR): 22.7% and partial response (PR): 63.6%). The 1-yr. local control rate was 55.56%. The 1-year OS was 50% and median survival was 11 months (range 2–46 months). In univariate analysis, Child-Pugh stage (p = 0.0056) and SBRT dose (p = 0.0017) were significant factors for survival. However, in multivariate analysis, SBRT dose (p = 0.0072) was the most significant factor, while Child-Pugh stage of borderline significance. (p = 0.0514). Acute toxicities were mild and well tolerated.

**Conclusion:**

This study showed that SBRT can be delivered safely to huge HCC and achieved a substantial tumor regression and survival. The results suggest this technique should be considered a salvage treatment. However, local and regional recurrence remain the major cause of failure. Further studies of combination of SBRT and other treatment modalities may be reasonable.

## Introduction

Hepatocellular carcinoma (HCC) is one of the most common malignancy worldwide, and the leading cause of cancer death in South and East Asia. In small HCC, hepatic resection and other nonsurgical treatment modalities have contributed to good survival [1,2]. However, treatment for huge HCC (≧ 10 cm in diameter) remain a challenge. At present, hepatic resection is regarded as the most available treatment of choice, provided the patient’s hepatic function reserved is acceptable for resection [3-7]. For unresectable huge HCC, TACE is an alternative, but the response rates are generally poor for large tumors [8,9]. After failure of TACE or patients unsuitable for TACE due to co-existing morbidities such as portal vein thrombosis or other vascular extension, no standard treatment is available, and various clinical trial have been tried, but to date survival benefits have been limited. And without any treatment these patient will not survive more than 3 months [10].

With the recent advancement of radiation therapy technology, Stereotactic body radiotherapy (SBRT) has proven its efficacy in the treatment of liver tumors. In the majority of recent studies, SBRT has been shown to achieve a high rate of local control with low toxicity in particular for small or ≦5 cm tumor [11-13]. But limited information is available regarding the use of SBRT for treatment of huge unresectable HCC. With the help of internal markers (fiducials) and synchrony tracking of tumor during respiration. SBRT with Cyberknife (Accuray Inc, Sunnyvale, CA, USA) allows more accurate application by reducing the error margin with reducing the amount of normal tissue exposure during treatment, enhancing the chance of treating larger tumor with limited normal liver available or tumor in close proximity to critical organs [14-17]. Furthermore, fractionated SBRT may have 3 times the biological effect of conventional fractionated radiation therapy [18,19].

This study retrospectively analyzed the outcomes of 22 patients with unresectable huge HCC with no other treatment options but with good liver function reserve and acceptable performance status treated with Cyberknife (Accuray Inc., Sunnyvale, CA) SBRT. We also attempt to determine survival, toxicity and response after SBRT. And hope these data would provide new hope to these patients who would otherwise be abandoned in terms of therapeutic options.

## Methods and materials

### Patients

Between January 2009 to November 2011, 22 patients with unresectable huge (≥10 cm) hepatocellular carcinoma (HCC) not suitable for other standard treatments were treated at out center with Cyberknife SBRT. Patient included in our study was based on the following criteria (1) Pathological confirmation of HCC; (2) At least one radiological image with the classic HCC feature of enhancement accompany by a level of serum tumor marker alpha fetoprotein (AFP) of >200 ng/ml or at least 2 radiological image (CT/MRI/Angiogram) with the classic imaging finding of HCC; (3) longest tumor diameter of ≥ 10 cm; (4) an ECOG performance status of ≤ 2. All patients with multiple extrahepatic metastases, Child-Pugh C, intractable ascites, tumor closely attached to esophagus, stomach, duodenum and bowel, normal liver volume of less than 700 cc were excluded from treatment.

Mandatory baseline examinations include dynamic magnetic resonance imaging (MRI) and or 3-phase computed tomography (CT) of liver, complete blood study, liver function test, hepatitis B, C antigen and virus titers, alpha-fetoprotein (AFP), and chest images were arranged. Patient with HbsAg positive or elevated hepatitis B virus titer was given prophylactic anti-retroviral therapy from the start of SBRT to at least 6 months after treatment in prevention of post-RT reactivation of HBV [20,21].

The characteristics and disease variables of the 22 patients at the time of radiation treatment are summarized in Table 1. The age ranges from 45–91 with a median age of 71, and male predominant. Tumors were mostly located in the right lobe. The mean maximum tumor diameter was 11.36 cm (range 10–18 cm) and solitary type tumor was the most frequent type.

**Table 1:** Patient and treatment characteristics (n = 22)

**Characteristics**	**Number of patients (%)**
Age	
≧60 y.o.	17(77.3)
<60 y.o.	5(22.7)
Gender	
Male	18(81.8)
Female	4(18.2)
ECOG	
0	3(13.6)
1	17(77.3)
2	2(9.1)
BCLC	
B	2(9.1)
C	20(90.9)
Vascular extension (PVTT & IVCTT)	
Yes	16(72.7)
No	6(27.3)
Child-Pugh stage	
A	20(90.9)
B	2(9.1)
Hepatitis virus	
B	12(54.5)
C	4(18.2)
Non B non C	6(27.3)
AFP	
<1500	15(68.2)
1500	7(31.8)
Tumor Location	
Right	21(95.5)
Left	1(4.5)
Tumor type	
Solitary	11(50)
Multiple	8(36.4)
Diffuse	3(13.6)
Tumor size	
10 cm	7(31.8)
>10 cm	15(68.2)
Radiation dose	
40 Gy	16(72.7)
< 40 Gy	6(27.3)
Pre-SBRT treatment	
Yes	9(40.9)
No	13(59.1)
Post – SBRT treatment	
Yes	6(27.3)
No	16(72.7)

The advantages and disadvantages of cyberknife SBRT were explained to the patients, and the final treatment depended on patients’ decisions. Written informed consent was obtained from all patients before treatment, and the study was approved by the institutional review board of Chi Mei Medical Center.

### SBRT

SBRT was performed using the Cyberknife, a robotic image-guided whole body radiosurgical system, equipped with the synchrony system, a real-time respiratory tracking system for target volumes that move with respiration. The total accuracy is less than 1.5 mm with Synchrony for mobile targets, with a treatment accuracy of 0.3 mm [22] 4 gold fiducial markers were implanted percutaneously around the perimeter of the target volume using a sono-guided procedure 5–7 days before the acquisition of the CT-scan used for planning. Thin-slice CT scan and MRI were performed with a slice thickness of 1 mm and transferred to the Cyberknife planning system. The contouring was performed on the planning CT with contrast. While the hepatic phase or delay phase of MRI was fuse with the planning CT-scan for contouring, other phases of MRI were also used as a visual reference. The PTV and organs at risk were delineated on the CT scan, and the system automatically performed an optimization of beam directions and beam weight in order to maximize the dose delivered to the target, minimizing the dose delivered to the organs at risk. All patients were positioned on individually shaped vacuum pillows and wore a treatment jacket on which the optical markers were fixed. Any displacement of the patient during treatment was detected by either internal or external fiducial markers with sub-millimeter accuracy [22].

### Dose specification and plan evaluation

Prescribed doses, doses per fraction and number of fractions were individualized based upon tumor size, location, amount of normal liver available and organ at risk. SBRT doses range from 26 Gy to 40 Gy in 5 fractions, with 40 Gy as the predominant prescribed dose, found in 16 patients. The gross tumor volume (GTV) included contrast-enhancing disease visible on CT scan or MRI with contrast. No CTV was further added. The GTV was directly expanded 1–3 mm in all direction to create the planning target volume (PTV). Modification of PTV was done if it extended into the dose-limiting organs, except the normal liver. Radiation doses were prescribed to the isodose line ranging from 59.9-96.9% of the maximum dose, median isodose line was 79.93%. Treatment was delivered with the real-time tracking system using the fiducial as a guide, planning with MultiPlan Cyberknife Treatment Planning System version 2.10.

The protocol dose constraints for normal liver (total liver minus cumulative GTV) specified that a minimum volume of 700 ml should receive a total dose less than 15 Gy [23], 66.7% of Ipsilateral right kidney volume should be less than 15 Gy, The maximum total dose to any point in the spinal cord should not exceed 18 Gy, and stomach, bowel, duodenum, heart should not exceed 30 Gy, while the esophagus should not be more than 27 Gy [24]. Efforts were made to minimize the dose to all normal tissues as low as possible.

### Follow-up, response, and toxicity assessment

After completion of treatment, the vital status evaluation, physical examination, liver function test, Complete blood test were followed to assess acute toxicity. They were followed every 1–2 weeks in the first months and every 3 months thereafter. Image study with 4-phase CT-scan or dynamic MRI of liver and AFP were followed 1–2 months and subsequently every 3- to 4-months. Toxicity grading was according to Common Toxicity Criteria Adverse Events version 4.0. Acute toxicities were defined as adverse events occurring within 3 months after SBRT, and late toxicities were those occurring after 3 months. Radiation-induced liver disease was defined as either classic or nonclassic RILD. Classic RILD was the presence of nonmalignant ascites and anicteric elevation of alkaline phosphatase level twice the upper normal level or baseline value occurring between 2 weeks and 3 months after the completion of irradiation. Nonclassic RILD, typically occurring between 1 week and 3 months after therapy, involves elevation of transaminase to at least 5 times the upper limit of the normal or pretreatment level within 4 months irradiation completion or decline in liver function in the absence of classic RILD [25-27]. This endpoint was common in HCC patients of poor liver function (hepatitis B infection, Child-Pugh Classic B and C) The diagnosis of both RILD could be made only in the absence of evidence of tumor progression. Toxicity grading was recorded based on the worst toxicity recorded.

Tumor response was assessed as described in the Response Evaluation and Criteria in Solid Tumors (RECIST) after completion of SBRT. A complete disappearance of the tumor was defined as complete response (CR), a decrease of more than 30% of the longest diameter of target tumor as partial response (PR), a decrease of less than 30% of the longest diameter of target tumor or no change as stable (SD), and progression of target tumor size of more than 20% as progressive disease (PD). Local recurrence was defined as an increase in tumor size or the development of a new lesion within the PTV. Regional recurrence was defined as the development of new lesion in non-targeted liver or outside the PTV. Distant metastasis was defined as recurrence beyond the liver, Disease progression was defined as the development of local recurrence, regional recurrence and distant metastasis.

### Statistical analysis

The Overall survival rate (OSR) and Disease-Progression free survival (DPFS) were estimated from the commencement of SBRT to the last follow-up using the Kaplan-Meier method. Univariate hazard ratio (HR) and 95% confidence interval were estimated by Cox proportional hazards regression model. Significant factors in univariate analysis were applied to the Multivariate Cox porportional hazard regression analysis. Analysis of data was performed using SPSS (SPSS Inc., Chicago, IL, USA) version 17 software. And the statistical significant level was set to p value <0.05.

## Results

### Tumor response and local control

**Figure 1:**
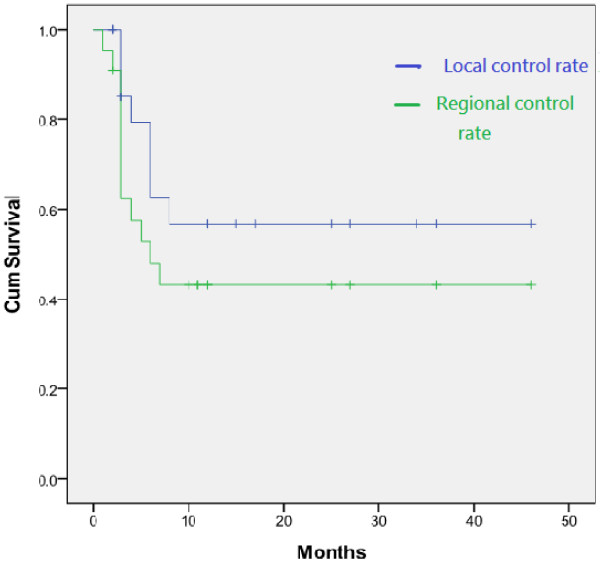
The 1-yr local control rate and regional control rate from Cyberknife SBRT.

The tumor response was based on the change in the maximum tumor size on serial CT-scans or MRI 4–8 weeks after completion of treatment and 2- to 3- months thereafter. After a median follow-up of 11.5 month (range 2–46 mo.), an objective response was achieved in 19 patients (86.3%), with complete response in 5 patients (22.7%), partial response in 14 patients (63.6%), and stable disease in 3 patients (13.6%) (Table 2). The 6 months local control rate was achieved in 11 patients (61.11%); 1-yr. local control rate in 10 patients (55.56%), and patients with a score of Child-Pugh A and those receiving dose of at least 40 Gy achieving a local control rate of 66.67% and 71.43%, respectively. While none was seen in Child-Pugh B patients and patient receiving dose of < 40 Gy. However, local recurrence and regional recurrence remain high, local recurrence rate at 6 months and 1-yr. were noted in 7 patients (38.89%) and 8 patients (44.45%), respectively. While regional recurrence was noted in 10 patients (47.62%) at 6 months and 11 patients at 1-yr. (53.31%) (Figure 1).

**Table 2:** Tumor response after SBRT (n = 22)

**Response**	**Patients (%)**
Complete	5 (22.7)
Partial	14 (63.6)
Stable	3 (13.6)

### Survival

**Figure 2:**
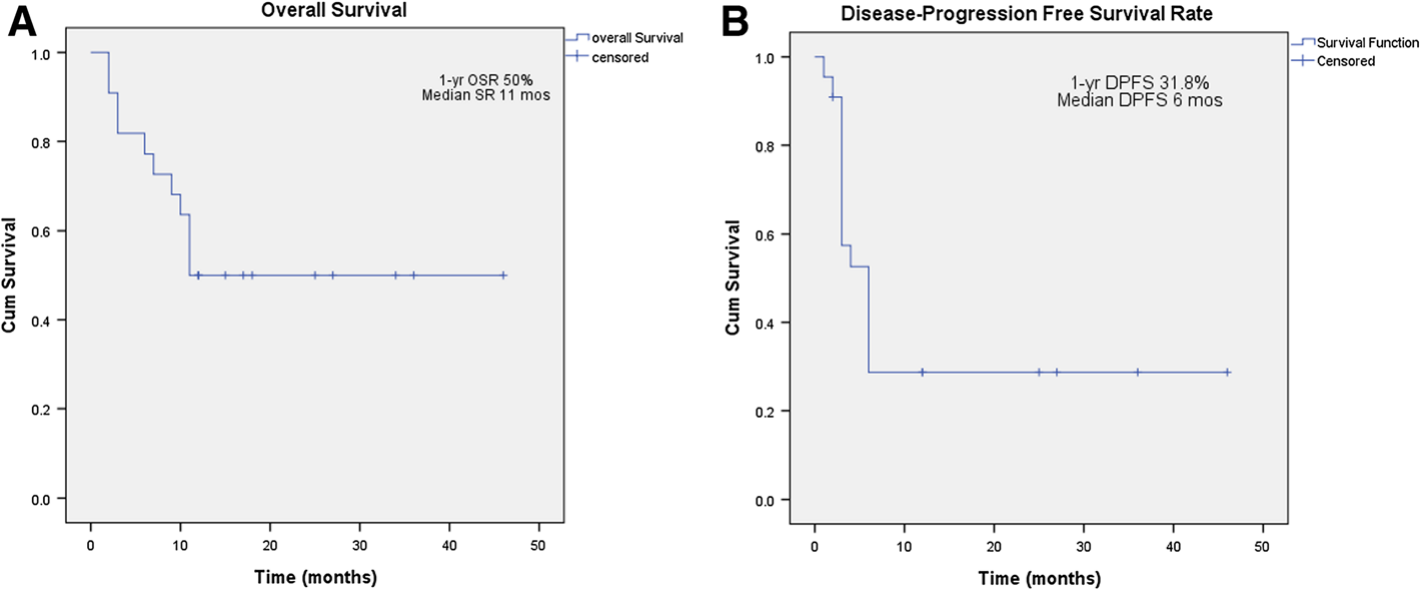
**(A) Overall survival rate and (B) Disease progression-free survival rate from Cyberknife SBRT.** The 1-yr. Overall survival rates and median survival time were 50% and 11 months, respectively. The 1 -yr. Disease progression-free survival rate and median disease-progression free survival rate were 31.8% and 6 months, respectively.

The median follow-up was 11.5 months after the start of SBRT. At the time of analysis, 11 patients had died and another 11 remained alive. The 6-months and 1-year OS was 81.8% and 50%, respectively, and median survival was 11 months (range 2–46 months) while 1-year disease progression-free survival rate was 31.8% and median time to disease progression was 6 months (range 2–46 months) (Figure 2). The 1-yr local control rate and regional control rate were 55.56% and 45%. And 1-yr. Overall survival rate for local control and regional control were 71.4% and 66.7%, respectively.

The analyses of the prognostic factors were based on survival calculations from the start of SBRT. In univariate analysis, Child-Pugh stage and SBRT dose were found to be significant prognostic factors for survival (Table 3). However, multivariate analysis showed that the SBRT dose was the most significant factor, while Child-Pugh stage was of borderline significance (Table 4).

**Table 3:** Prognostic factors affecting survival on univariate analysis

**Factor**	**1-yr. survival rate (%)**	**Hazard ratio (95% confidence interval)**	**p**
Age			
≧60 y.o.	41.2	1.293(0.343-4.882)	0.7
< 60 y.o.	60		
Gender			
Male	44.4	0.427(0.054-3.344)	0.42
Female	55.6		
ECOG			
0	66.7		
1	47.1	1.656(0.209-13.085)	0.63
2	50	2.155(0.134-34.555)	0.59
BCLC			
B	50		
C	50	0.915(0.117-7.176)	0.93
Vascular extension (PVTT & IVCTT)			
Yes	37.5	4.920(0.628-38.559)	0.13
No	83.3		
Child-Pugh score			
A	55		
B	0	17.303(2.301-130.129)	0.01
Hepatitis virus			
B	50		
C	25	1.938(0.483-7.774)	0.35
Non B non C	66.67	0.517(0.104-2.569)	0.42
AFP (IU/mL)			
<1500	60		
≧1500	28.6	2.920(0.869-9.809)	0.08
Tumor location			
Right	52.38		
Left	0	7.629(0.783-74.297)	0.08
Tumor type			
Solitary	54.6		
Multiple	50	1.447(0.387-5.410)	0.58
Diffuse	33.3	2.895(0.554-15.129)	0.21
Tumor size			
10 cm	57.4		
>10 cm	46.67	1.252(0.331-4.729)	0.74
Radiation dose			
40 Gy	68.75		
< 40 Gy	0	7.922(2.180-28.780)	0.01
Pre-SBRT treatment			
Yes	66.7		
No	38.5	2.576(0.679-9.777)	0.16
Post-SBRT treatment			
Yes	66.7		
N0	43.8	2.056(0.443-9.542)	0.36

**Table 4:** Multivariate analysis of poor prognostic factors

**Variables**	**Hazard ratio**	**95% confidence interval**	**p-value**
Child -Pugh stage			
A vs B	7.45	0.988-56.1	0.0514
Radiation dose			
40 Gy vs < 40 Gy	6.57	1.665-25.91	0.0072

### Toxicity

Toxicity was summarized in Table 5. No grade 4 or 5 toxicity occurred. In term of acute toxicities, Grade 1 fatigue and grade 1–2 thrombocytopenia were the most common sequelae, in 91% and 67% of the patients, respectively. 1 patient had grade 3 elevation of SGPT and 1 with grade 2 elevation of alkaline phospatase, but both were documented with regional and local recurrence. 4 patients have grade 1 elevation of alkaline phospatase, 1 was documented with regional recurrence and bone metastasis, 3 were mild elevation from the upper limit of less than 2 fold, Others liver function alterations were usually mild ranging from grade 1 to 2. The effects were transient and stabilized within 1–2 weeks. All patients completed SBRT without interruption due to intolerable side effect. Fortunately, no patients suffer from severe RILD nor death related to treatment. Grade 1–2 rib pain and local skin induration of right lateral abdominal wall were in 5 and 3 patients, respectively. No Gastrointestinal toxicity such as gastroduodenal ulcer, gastroenteritis nor colitis were noted. Since most of the treated tumors (21) were located in the right lobe of the liver, dose to bowel and stomach were minimal, and the 1 patient treated in the left lobe, strict dose constraint to the bowel and GI tract. were adhered. While for patients with huge HCC tumor closely adherence to the ribs, inorder not to compromise tumor coverage, rib volume was not constraint. Nonetheless, neither rib fracture nor skin ulceration was observed.

**Table 5:** Toxicity grade observed after SBRT (n = 22)

**Adverse events**	**Toxicity grade**			**Total no. (%)**
	**1**	**2**	**3**	
Acute toxicity				
SGOT	8	0	0	8(36.4)
SGPT	7	2	1	9(40.1)
Total Bilirubin	0	1	0	1(4.5)
Alk.phospatase	4	2	0	6(27.27)
Albumin	8	2	0	10(45.4)
Platelet	13	1	0	14(63.6)
Fatigue	20	0	0	20(90.1)
Nausea	11	0	0	11(50)
Vomiting	1	0	0	1(4.5)
Radiation dermatitis	2	1	0	3(13.6)
Rib pain	3	2	0	6(27.3)
Skin induration	2	1	0	3(13.6)

## Discussion

During the past decade, a number of reports have documented the effect of SBRT on HCC. There remain no optimal dose and fractionation scheme, but the current consensus stated high dose local RT alone or combine with other modalities such as TACE could achieve a high rate of local control [28-30] However, these reports were mostly limited to smaller tumors or tumor less than 10 cm [31-36]. For Huge HCC, it remain a challenging role for radiation therapy, because of close proximity to critical organ, limited liver volume available and a relatively poor liver functional status. At present there are only scarce studies on this issue. In our study, our preliminary results support the fact that SBRT could be an alternative treatment for unresectable huge HCC with no other treatment option. 1-yr OS of 50% is comparable to that of TACE for tumor >5 cm [3,31]. At present, TACE was the most common alternative treatment for inoperable large HCC. Although long-term survival has been reported after TACE, but reported tumor response is achieved in only 17–61% and complete response is rare because of viable tumor cells remain after TACE [8]. After failure of TACE, other treatment options such as target therapy, hepatic intraarterial chemotherapy were unsatisfactory for large HCC. And thus, our present study resulted in a 1-yr. objective response rate of 86.3% (CR, 22.7% and PR, 63.6%). 1-yr. local control rate of 55.56% (Child-Pugh A 66.67%, Child-Pugh B 0%; Radiation dose 40 Gy 71.43%, dose <40 Gy 0%) and a median survival of 11 months (range 2–46 months) were encouraging. Accordingly, we view Cyberknife SBRT as one of the best alternative treatment modality for inoperable huge HCC patient particular for Child-Pugh A patients and those tolerating a radiation dose of at least 40 Gy.

Our present trial was too small to allow stratification by prognostic factors, however, exploratory multivariate analysis showed that a higher radiation dose (40Gy/5 fractions) independently predicted overall survival. Several studies have shown a dose–response relationship between conventional radiation therapy dose and response in HCC. And higher more intense SBRT dose contribute to higher local control rates [32-36]. Seo et al. [35] prospectively studied 38 patients with inoperable HCC (<10 cm) treated by Cyberknife SBRT, radiation doses range from 33–57 Gy in three to four fractions were prescribed according to tumor volumes. The local response rate was 63% at 3 months after SBRT. And two-year overall survival and local progression-free survival rates were 61.4% and 66.4%., a high radiation dose was found as the independent prognostic factor. While Rusthoven et al. [36], In a phase II study, using 60 Gy in three fractions, resulted in an actuarial in-field local control rate for liver metastasis (< 6 cm) at 1 and 2-yrs after SBRT were 95% and 92%, respectively. Moreover, for maximal diameter of 3 cm or less, 2-yr. Local control rate was 100% and only one case of grade 3 soft tissue toxicity was observed. Hence, high-dose liver SBRT was safe and effective when normal liver tissue constraint was met. Chang et al. [37], recently studied SBRT for colorectal liver metastases in 3 major institutions. The study demonstrates that local control is dose dependent, with a 18-month local control of 84% for total doses ≥ 42 Gy versus 43% for total doses <42 Gy. Overall, the total dose, dose per fraction and biological effective dose were significant in univariate and multivariate analysis. Nevertheless, it must be reminded that most HCC usually associated with liver cirrhosis and poor liver functions was more susceptible to liver toxicity rather than liver metastasis. In our present study, we strictly specified that a minimum volume of 700 cc of normal liver should receive a total dose less than 15 Gy.

Compared with other Radiation therapy technique, Cyberknife SBRT with respiratory synchrony tracking system demonstrated its advantage in treating huge HCC with a limited normal liver preserved. These systems are demonstrably more conformal and able to minimize radiation outside the PTV, thus sparing critical structures near the tumor than those generated by other system. This also delivered higher biological effective doses without increasing incidence of liver toxicity incidences, and attained a higher local control rate.

In our present study, other than transient hepatic function disorders and gastrointestinal toxicities commonly observed. Neither grade 3 complications nor radiation-induced liver disease (RILD) were observed. There were 5 patient suffered from Grade 1–2 painful musculoskeletal complication, notably right lower rib pain and skin induration, all these patients have huge HCC closely adherent to adjacent ribs and skin. In order not to compromise the PTV coverage, rib and skin constraints were not considered, but fortunately, no severe complication above grade 2 was observed. Although musculoskeletal complications were not life-threatening event,these morbidity should be considered in treating huge HCC with high SBRT dose. In the recent multicenter analysis by Benedict et al. [24] reported SBRT doses in 5 fractions, the maximum point dose of 43 Gy (8 Gy/fx) on rib volume should be less than 1 cc. And less than 10 cc of skin volume should not receive more than a max point dose of 39.5 Gy (7.9 Gy/fx). In another study by Dunlap et al. [38], recommended Chest wall volume receiving 30 Gy in three or five fractions should be limited to <30 cc. to reduce toxicity without compromising tumor coverage.

Huge HCC have shown to harbor microvessel tumor invasion, poor differentiation, a propensity for multinodular lesions and subsequent recurrence. A Recurrence rate of more than 70% after resection of very large HCC (>10 cm) was reported by Shah et al. [8]. In our study, regional and local recurrences remain the major pattern of failure. Thus, combining SBRT with other treatment modality with non-overlapping toxicity such as TACE, target therapy, and other potential drugs with anititumor effects on HCC in clinical studies may potentially increases local control, decrease regional failure and prolonged survival rates.

The major limitation of this study is that it was a retrospective single-institution study with small sample size, the median survival rate was only 11 months, which is most likely related to the unfavorable prognostic features of the patient enrolled. Furthermore, late toxicity may be underestimated as a result of limited survival, especially when the higher than conventional fractional dose is considered.

## Conclusions

In conclusion, despite its unfavorable prognosis, our study supports that Cyberknife SBRT is feasible in treating huge unresectable HCC, tumor response rate of 86.3% (CR + PR), 1-yr. local control rate of 55.56%. And the 1-yr. Overall survival rate of 50% were encouraging. While patients with a score of Child-Pugh A and those receiving doses of at least 40 Gy were able to achieved a 1-yr local control rate of 66.67% as well as 71.43% were promising Acute toxicities were mild and tolerable. However, local and regional recurrence remained the major problem. Prospective studies of combination of SBRT with other treatment modalities may be suggested.

## Competing interests

The authors declare they have no competing interests.

## Authors’ contributions

JQ reviewed, analyzed, interpreted the data, and wrote the manuscript. LCL, KLL, CHL, YWL, CCY provided significant intellectual contribution and reviewed the manuscript. All authors gave the final approval of the manuscript’s submission for publication.
